# MicroRNA-133 overexpression promotes the therapeutic efficacy of mesenchymal stem cells on acute myocardial infarction

**DOI:** 10.1186/s13287-017-0722-z

**Published:** 2017-11-25

**Authors:** Yueqiu Chen, Yunfeng Zhao, Weiqian Chen, Lincen Xie, Zhen-Ao Zhao, Junjie Yang, Yihuan Chen, Wei Lei, Zhenya Shen

**Affiliations:** 1 Institute for Cardiovascular Science & Department of Cardiovascular Surgery of The First Affiliated Hospital, Soochow University, 215007, 708 Renmin Rd, Bldg 1, Suzhou, China; 2 Nantong First People’s Hospital, 226001, North Rd, Haier alley, Nantong, China

**Keywords:** miR-133, Mesenchymal stem cells, Myocardial infarction

## Abstract

**Background:**

Our study aim was to evaluate the therapeutic efficacy and mechanisms of miR-133-overexpressing mesenchymal stem cells (MSCs) on acute myocardial infarction.

**Methods:**

Rat MSCs were isolated and purified by whole bone marrow adherent culturing. After transfection with the agomir or antagomir of miR-133, MSCs were collected for assay of cell vitality, apoptosis, and cell cycle progression. At the same time, exosomes were isolated from the supernatant to analyze the paracrine miR-133. For in-vivo studies, constitutive activation of miR-133 in MSCs was achieved by lentivirus-mediated miR-133 overexpression. A rat myocardial infarction model was created by ligating the left anterior descending coronary artery, while control MSCs (vector-MSCs) or miR-133-overexpressed MSCs (miR-133-MSCs) were injected into the zone around the myocardial infarction. Subsequently, myocardial function was evaluated by echocardiography on days 7 and 28 post infarction. Finally the infarcted hearts were collected on days 7 and 28 for myocardial infarct size measurement and detection of *snail 1* expression.

**Results:**

Hypoxia-induced apoptosis of MSCs obviously reduced, along with enhanced expression of total poly ADP-ribose polymerase protein, after miR-133 agomir transfection, while the apoptosis rate increased in MSCs transfected with miR-133 antagomir. However, no change in cell viability and cell-cycle distribution was observed in control, miR-133-overexpressed, and miR-133-interfered MSCs. Importantly, rats transplanted with miR-133-MSCs displayed more improved cardiac function after acute myocardial infarction, compared with those that received vector-MSC injection. Further studies indicated that cardiac expression of *snail 1* was significantly repressed by adjacent miR-133-overexpressing MSCs, and both the inflammatory level and the infarct size decreased in miR-133-MSC-injected rat hearts.

**Conclusions:**

miR-133-MSCs obviously improved cardiac function in a rat model of myocardial infarction. Transplantation of miR-133-overexpressing MSCs provides an effective strategy for cardiac repair and modulation of cardiac-related diseases.

**Electronic supplementary material:**

The online version of this article (doi:10.1186/s13287-017-0722-z) contains supplementary material, which is available to authorized users.

## Background

Myocardial infarction (MI), arising from myocardial ischemia, is the leading cause of morbidity and mortality in the world [1]. In the process of MI and subsequent heart failure, the myocardium undergoes structural alterations, such as apoptosis of myocardial cells, increase of extracellular matrix protein, fibrosis, and hypertrophy of cardiac myocytes [2].

Given their multipotency, low immunogenicity, availability, and capacity for expansion, mesenchymal stem cells (MSCs) are being considered as a potential cell source for tissue repair and have been used in clinical trials to cure intractable diseases [3, 4]. In patients with severe ischemic heart failure, MSC transplantation could reduce the left ventricular end-systolic volume (LVESV) and improve the left ventricular ejection fraction (LVEF), stroke volume, and myocardial mass. What is more important is that no side effect was found in these clinical trials [4, 5]. To gain more therapeutic effect, continued efforts are emerging to improve cardioprotective effects of MSCs, using the methods of exogenous stimulation or endogenous genetic engineering. Overexpression of *CXCR4* can enhance the mobilization and engraftment capacity of MSCs to the ischemic area, promote the secretion of paracrine factors, and enhance neovascularization [6–9]. Except for their multilineage differentiation ability and paracrine effects, MSCs have been shown to ameliorate lipopolysaccharide (LPS)-induced inflammation in a rat model of acute lung injury (ALI) [10].

MicroRNAs (miRNAs) are 19–22-nucleotide nonprotein-coding RNAs (ncRNAs) which are expressed ubiquitously and induce mRNA degradation or inhibit translation by direct binding to the 3′ untranslated region (3′UTR) of their target mRNAs [11, 12]. miRNAs have been reported to modulate cardiac proliferation, differentiation, survival, autophagy, pyroptosis, and reprogramming [13–15]. Prabhu et al. [16] demonstrated a crucial influence of miRNAs in the inflammation and regeneration phases of cardiac repair after MI. miR-21 reduced the H_2_O_2_-induced apoptosis of BMSCs through downregulating the *PTEN* gene and PI3K/Akt pathway. miR-133 was recently shown to be involved in the process of apoptosis, vascular smooth muscle cell differentiation, angiogenesis, and regeneration of cardiomyocytes [17]. The abundance of miR-133 is higher in healthy adult hearts when compared with that in fetal hearts. However, cardiac expression of miR-133 significantly decreased in patients suffering from MI [18]. Elevation of miR-133 reduced hypoxia-induced, oxidative stress-induced, and endoplasmic reticulum stress-induced cardiac apoptosis in vitro [19–21]. What is more, miR-133 was reported to affect β-adrenergic receptor signaling and inhibit cardiac hypotrophy in the process of heart failure [22, 23]. Regulatory exercise can prevent cardiac cell apoptosis through accelerating the expression of miR-133 and *Bcl-2* [24].

The aim of this study was to elucidate the effects of miR-133 on the function of MSCs in treating ischemic myocardial diseases. We undertook both overexpression and knockdown approaches to demonstrate the anti-apoptotic role of miR-133 on MSC survival and the cardioprotective role of miR-133-MSCs in infarcted hearts. The study provides a novel therapeutic approach showing a better therapeutic effect on MI.

## Methods

### Animals

All animal experiments were approved by the Ethic Committee of Soochow University (reference number: SZUM2008031233) and were conducted according to institutional animal ethics guidelines for the Care and Use of Research Animals established by Soochow University, Suzhou, China. Forty rats were purchased from the Laboratory Animal Center of Nanjing University (Nanjing, China) and maintained under specific pathogen free conditions.

### Isolation, culture, and characterization of bone marrow MSCs

Bone marrow-derived mesenchymal stem cells (BM-MSCs) were isolated and cultured as described previously [25]. Briefly, bone marrow was flushed from femora of SD rats. Cells were collected by centrifugation, seeded onto a culture dish, and cultured in DMEM/F12 supplemented with 10% fetal bovine serum (FBS). The culture medium was refreshed every 48 h and the colonies of MSCs should appear in 8–10 days. The phenotype of MSCs was identified by flow cytometry at passage 4, using antibodies against rat CD90-APC, CD11b/c-FITC, and CD45-PEcy7.

### Hypoxia conditioning of MSCs in vitro

For hypoxic culture, MSCs were cultured in a tri-gas incubator (Thermo Fisher Scientific, Marietta, OH, USA) composed of 94% N_2_, 5% CO_2_, and 1% O_2_. To determine the optimal duration of hypoxic treatment, cells were cultured for 6, 12, 24, and 48 h under hypoxic and normoxic conditions. MSCs were then harvested for flow cytometric analysis of apoptosis and proliferation.

### Transient transfection

MSCs (3 × 10^5^ cells) were seeded onto the 12 wells of a 12-well plate, incubated overnight, and then transfected with miR-133a agomir, miR-133a antagomir, or negative control using Lipofectamine 2000 (Invitrogen) according to the manufacturer’s instructions as described previously [3].

### Quantitative RT-PCR assay

Total RNA was isolated from MSCs or left ventricular tissues using Trizol reagent (Invitrogen, USA) as described previously [3]. Subsequently, RNAs were reverse-transcribed to cDNAs with the microRNA reverse transcription system (GenePharma, Shanghai, China) or the primescript RT reagent kit (TAKARA, Japan). The expression level of miR-133a was analyzed by quantitative RT-PCR (Q-PCR) using the miRNAs Quantitation Kit (GenePharma, China). For *snail 1*, Q-PCR was performed using SYBR PCR master mix in the ABI Step One-Plus Detection system (Applied Biosystems, USA) according to the manufacturer’s introductions. The primers used for *snail 1* are as follows: sense, 5′-TTCTCCCGAATGTCCTTGCT-3′; and antisense, 5′-CTGCCTTCCATCAGCCATCT-3′. Relative gene expression quantifications were calculated according to the comparative Ct method using U6 as an internal standard. Each assay was performed in triplicate.

### Viability and proliferation assay

Cell viability was evaluated by CCK-8 assay (Dojindo Laboratories, Japan) as described previously [26]. Briefly, MSCs in 96-well plates were transfected with miR-133 agomir/antagomir and cultured for 6, 12, 24, and 48 h. The culture supernatants were then changed to fresh medium with 10% CCK-8 (Dojindo Laboratories, Japan). The absorbance at 450 nm indicating cell viability was measured in the Multi-Mode Microplate Reader (BIOTEK, USA), taking into account the background readings. The proliferation of MSCs was assessed with carboxyfluorescein diacetate succinimide ester (CFSE) (Invitrogen). MSCs were stained with CFSE at a concentration of 25 mM for 10 min, washed with PBS three times, and transfected with miRNAs in 12 h. MSCs were then collected at 6, 12, 24, and 48 h and analyzed by flow cytometry (Guava).

### Flow cytometry

Cell cycle and apoptosis were analyzed by flow cytometry as reported previously [3]. MSCs at passage 5 were placed in a 12-well plate at a confluence of 80% and then transfected with miR-133 agomir/antagomir or negative controls. At corresponding time points, cells were collected and stained with Annexin-V (eBioscience, USA) and propidium iodide (for apoptosis assay) or only propidium iodide (Sigma, for cell cycle assay) for 30 min. The cells were detected by flow cytometry, and data were analyzed by FlowJo software.

### Reconstruction of plasmids and preparation of lentivirus

The vector pCDH-CMV-MCS-EF1-copGFP was used as a backbone plasmid to reconstruct lentiviral vector containing miR-133a. The vector and pre-miR-133a fragments amplified from rat genomic DNA were digested with *Xba*I and *Eco*RI (NEB, USA) and ligated with T4 ligase (TAKARA). The primers used for amplification of pre-miR-133a are as follows: sense, 5′-ATGTCTAGACCCTCTAATACTCGTCAT-3′; and antisense, 5′-GAATTCGACCGTTGTTAGTTGTTT-3′. The reconstruct plasmid was verified by Sanger sequencing. For lentiviral production, HEK293NT cells were cotransfected with control vector or lentiviral plasmid carrying pre-miR-133 fragments, along with lentiviral packaging mix. The culture medium was collected at 24 and 48 h after transfection, filtered through a 0.45-μm filter, and incubated overnight with polyethylene glycol 8000 (PEG 8000) before being concentrated by centrifugation (4000 × *g* for 20 min at 4 °C; Thermo). Lentivirus was titrated using a titer kit, and virus particles were stored at –80 °C until use. The ratio of lentivirus-infected MSCs was observed under an inverted fluorescence microscope (Olympus, Tokyo, Japan).

### Rat model of acute MI and assessment of heart functions

Acute MI was induced as described previously [27, 28]. Briefly, young Sprague–Dawley rats (~250 g) were anesthetized with 10% chloral hydrate (w/v; Acros, Japan) by intraperitoneal injection. The left anterior descending artery was ligated between the pulmonary artery outflow tract and the left atrium. While sham-ligated rats were used as control, these rats under MI surgeries were divided into four groups receiving intramyocardial injection of PBS, normal MSCs, MSCs infected with empty lentivirus (vector-MSCs), or MSCs infected with miR-133-overexpressed lentivirus (miR-133-MSCs), respectively. A total of 1 × 10^6^ MSCs in 20 μl of phosphate buffer (PBS) were transplanted by myocardial injection near the ligation site in the free wall of the left ventricle. Rats were anesthetized for echocardiography detection on days 0, 7, and 28 after MI, using the Vevo 2100 system (VisualSonics Inc., Toronto, ON, Canada) with an 80-MHz probe. Rats were finally sacrificed to harvest the heart tissue for Q-PCR analysis, hematoxylin and eosin (H&E) staining, and Masson staining.

To evaluate the effect of miR-133 on cell survival in vivo, normal MSCs, MSCs infected with empty lentivirus (vector-MSCs), or miR-133-overexpressed lentivirus (miR-133-MSCs) were dyed with chloromethylbenzamido (CellTracker^TM^ CM-Dil 11372053; Invitrogen) according to the manufacturer’s instructions, followed by cell transplantation. Rats were anesthetized for MSC survival detection on day 3 after MI, and CM-Dil^+^ MSCs were observed via fluorescence microscopy (Olympus, Japan).

### Histological examination

Freshly isolated hearts were fixed in 4% paraformaldehyde (PFA, pH 7.4) and sectioned. H&E and Masson trichrome staining were performed to demonstrate myocardial fibrosis as described previously [3]. For Masson trichrome staining, heart slices were produced perpendicular to the axis of the left anterior descending coronary artery. The severity of myocardial fibrosis was indicated by the average ratio of the fibrotic area to the entire LV cross-sectional area and the average ratio of fibrosis length to entire internal LV circumference, which was analyzed by ImageJ software (National Institutes of Health, USA).

### Exosome isolation

MSCs were transfected with miR-133 agomir, miR-133 antagomir, or negative control, and subsequently cultured in DMEM/F12 with exosome-free FBS. Exosomes from culture supernatants were isolated by differential centrifugation. In brief, supernatants were centrifuged at 200 × *g* for 30 min to remove cell debris (991; Thermo). Subsequently, the supernatants were mixed with total exosome isolation reagent (Invitrogen, USA) overnight at 4 °C. After centrifuging at 10,000 × *g* for 1 h, the pellet was resuspended in PBS. miRNA in the exosome was isolated using a mirVana miRNA isolation kit (Invitrogen, USA) according to the manufacturer’s instructions. Subsequently, the expression level of miR-133a was analyzed by Q-PCR as already described. The protein level in the exosome was analyzed by BCA assay kit (Beyotime, China) and the secreted level of CD63 was detected by western blot analysis as described in the following.

### Coculture of MSCs with cardiomyocytes

Cardiomyocytes were isolated from neonatal rat with 1 mg/ml collagenase II (Invitrogen, USA). Forty-eight hours later, the isolated cardiomyocytes were cocultured with MSCs transfected with miR-133 agomir/miR-133 antagomir or negative control at a 10:1 ratio. After 48 h of coculture, the cardiomyocytes were collected for RNA isolation and Q-PCR analysis.

### Western blot analysis

Western blot analysis was performed according to previous description [27]. Total protein (30 μg protein per lane) from MSCs transfected with different miRNAs was subjected to SDS-PAGE and immunoblotting with the antibody against poly ADP-ribose polymerase (PARP) (46D11; CST), CD63 (ab108950; Abcam), Collagen I (14695; Proteintech), α-SMA (ab5694; Abcam), and GAPDH (KM9002T; Sungene Biotech).

### Statistical analysis

Data were expressed as mean ± SEM. Statistical analysis was performed using one-way ANOVA to compare data among three or more groups and Student’s unpaired *t* test to compare data between two groups. *p* < 0.05 was considered statistically significant. Statistical analysis was carried out using GraphPad Prism software (version 5.01; San Diego, CA, USA).

## Results

### Characterization of rat MSCs

**Fig. 1:**
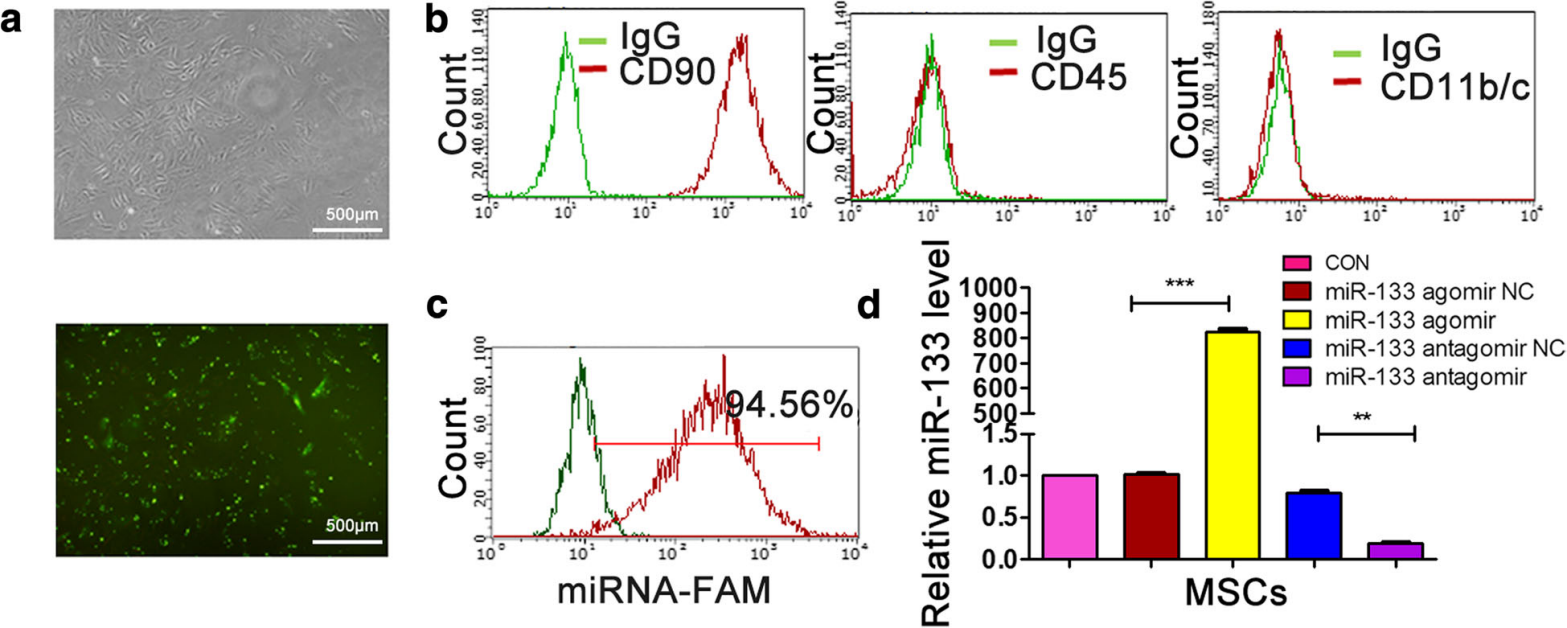
Characterization of bone marrow-derived mesenchymal stem cells (MSCs) transfected with miR-133. **a** Morphology of MSCs observed under inverted fluorescence microscope (upper panel, bright field; lower panel, fluorescence field). **b** Phenotypic analysis of cell surface antigens of MSCs by flow cytometry. **c** Transfection efficiency detected by flow cytometry. **d** miR-133 expression level determined by Q-PCR. **p < 0.01, ***p < 0.001. Data representative of three independent experiments. CON control, NC negative control

MSCs were isolated from bone marrow of SD rats. MSCs were typically spindle-shaped and adherent to the plastic dishes (Fig. 1a). The surface markers of MSCs were further identified by flow cytometry. Results showed that BM-MSCs were positive for CD90, and negative for CD45 and CD11b/c (Fig. 1b).

In order to determine whether miR-133 affected MSCs, we transfected MSCs with miR-133 agomir/antagomir. The transfection efficiency was evaluated with FAM-labeled nucleic acid controls (Fig. 1a, c) and 94.56% of MSCs were positive for FAM signal by flow cytometry analysis. Q-PCR revealed that transfection of miR-133 agomir elevated miR-133 expression in MSCs by 800-fold when compared with the negative control, and miR-133 antagomir reduced miR-133 expression by 80% (Fig. 1d). These data confirmed that MSCs were successfully isolated and modulated with miR-133 agomir/antagomir.

### miR-133 enhanced survival of MSCs under hypoxic conditions

To investigate the role of miR-133 in the survival of MSCs, we evaluated apoptosis, cell vitality, and cell cycle in miR-133 agomir/antagomir-transfected MSCs under hypoxic conditions.

**Fig. 2:**
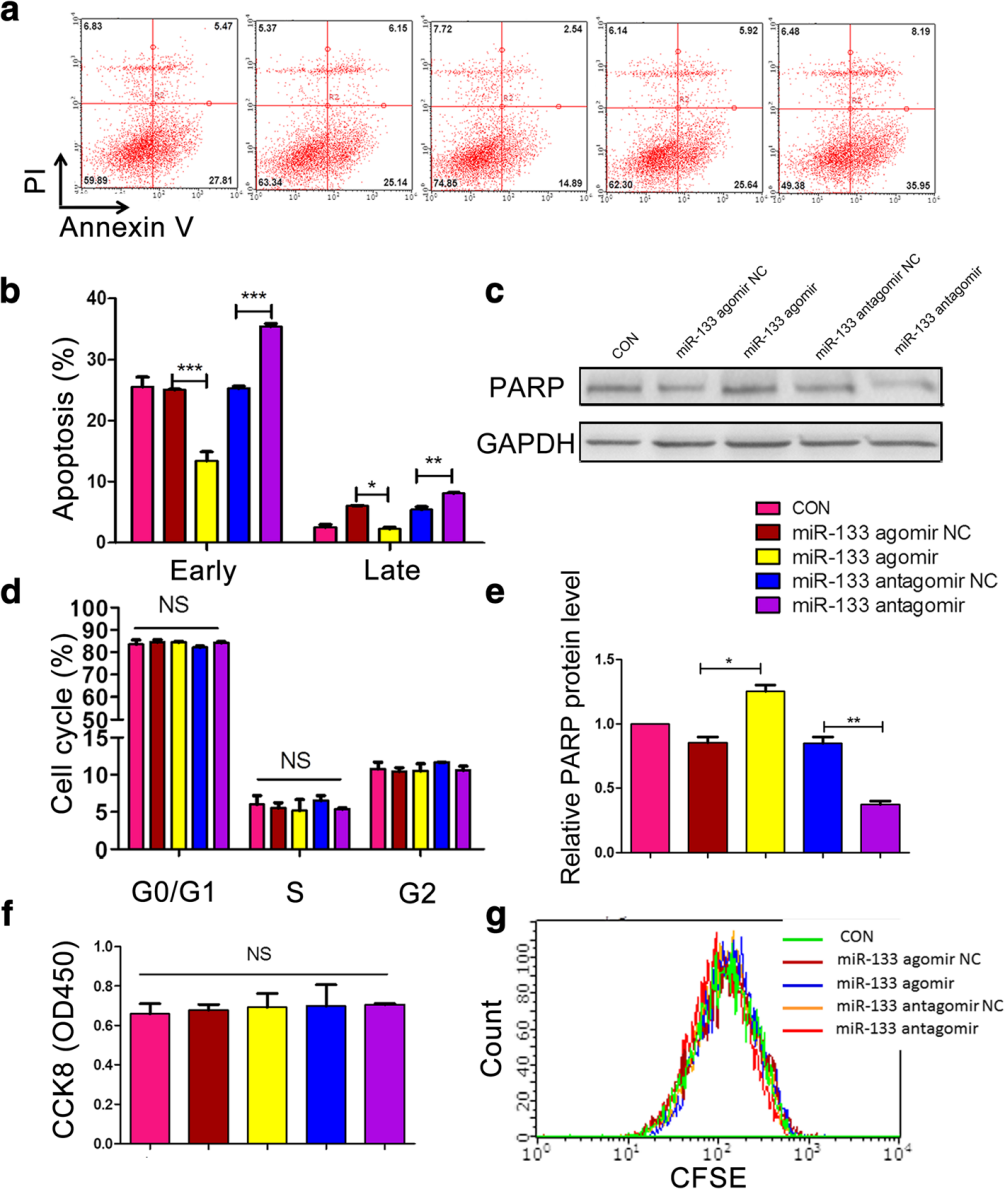
miR-133 enhanced the survival of MSCs under hypoxic conditions. Apoptosis analyzed by Annexin V/PI staining and flow cytometry. **a** Scatter diagram of apoptosis in MSCs transfected with miR-133 agomir, antagomir, or their corresponding negative control (NC). **b** Histogram of apoptosis in different groups. **c** Western blot analysis of PARP protein in MSCs transfected with different miRNAs. **d** Cell cycle analyzed by PI staining and flow cytometry. **e** Histogram of western blot data from (**c**). **f** Cell viability detected by CCK8 assay (*n* = 5). **g** Cell proliferation detected using CFSE at 24 h after transfection with miRNAs. *p < 0.05, **p < 0.01, ***p < 0.001. CFSE carboxyfluorescein diacetate succinimide ester, CON control, NS not significant, PARP poly ADP-ribose polymerase, PI propidium iodide

We first performed Annexin V/PI staining, followed by flow cytometry, to detect the effect of hypoxic conditions on cell survival. When cultured under low oxygen conditions, the rate of MSC apoptosis increased in a time-dependent manner, and reached the maximum ratio at 24 h (Additional file 1: Figure S1A). Thus, we chose 24-h treatment to further study the influence of miR-133 on hypoxia-induced MSC apoptosis. Our study demonstrated that both early and late apoptosis were obviously decreased in MSCs transfected with miR-133a agomir. On the contrary, miR-133a antagomir significantly facilitated hypoxia-induced MSC apoptosis (Fig. 2a, b). PARP is a typical apoptosis related protein cleaved by caspase 3. We found that the full-length protein level of PARP was elevated in the miR-133 agomir group and downregulated in the miR-133 antagomir group (Fig. 2c), indicating that miR-133 decreased caspase 3 activity in MSCs.

However, no obvious change in cell-cycle distribution (Fig. 2d), cell viability (Fig. 2f and Additional file 1: Figure S1B), and cell proliferation (Fig. 2g and Additional file 1: Figure S1c) was detected in MSCs transfected with control miRNA, miR-133 agomir, or miR-133 antagomir.

### MSC infection with miR-133a lentivirus

**Fig. 3:**
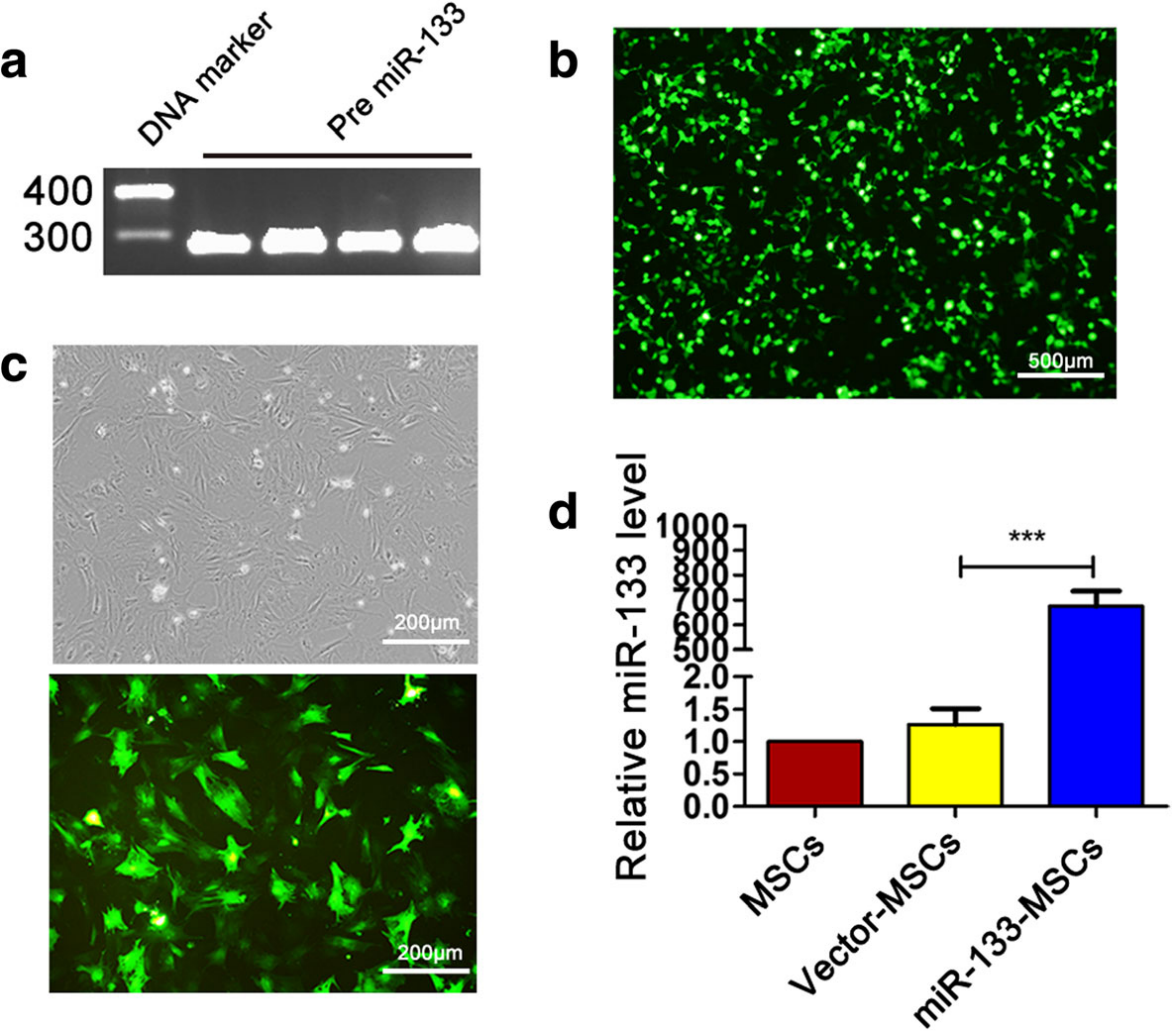
Construction of miR-133 lentivirus. **a** Electrophoretogram of pre-miR-133 fragments. **b** Fluorescence image of GFP^+^ 293NT infected with lentivirus carrying pre-miR-133. **c** Morphology of MSCs transfected with miR-133 lentivirus (upper panel, bright field; lower panel, fluorescence field). **d** miR-133 expression level detected by Q-PCR after lentiviral transfection of miR-133. ***p < 0.001. MSC mesenchymal stem cell

We extracted the miR-133a and pre-miR133 sequences of rats in miRBase (http://www.mirbase.org/). Fragments of rat pre-miR-133 (281 bp) were amplified by PCR from rat genomic DNA (Fig. 3a). The amplified fragments were cloned into the multiple cloning site (MCS) of pCDH-CMV-MCS-EF1-copGFP. The fusion plasmid was then cotransfected into 293NT cells along with a lentivirus package mix (Fig. 3b). An empty backbone vector was used as a control.

After lentiviral infection, most MSCs expressed green fluorescent protein (GFP), suggesting successful lentiviral packaging and cell infection (Fig. 3c). As shown in Fig. 3d, the miR-133 level was increased by 700-fold in MSCs infected by miR-133-overexpressing lentivirus, when compared with that in both uninfected and vector-lentivirus-infected MSCs.

### miR-133-MSCs effectively preserve cardiac function in a rat MI model

**Fig. 4:**
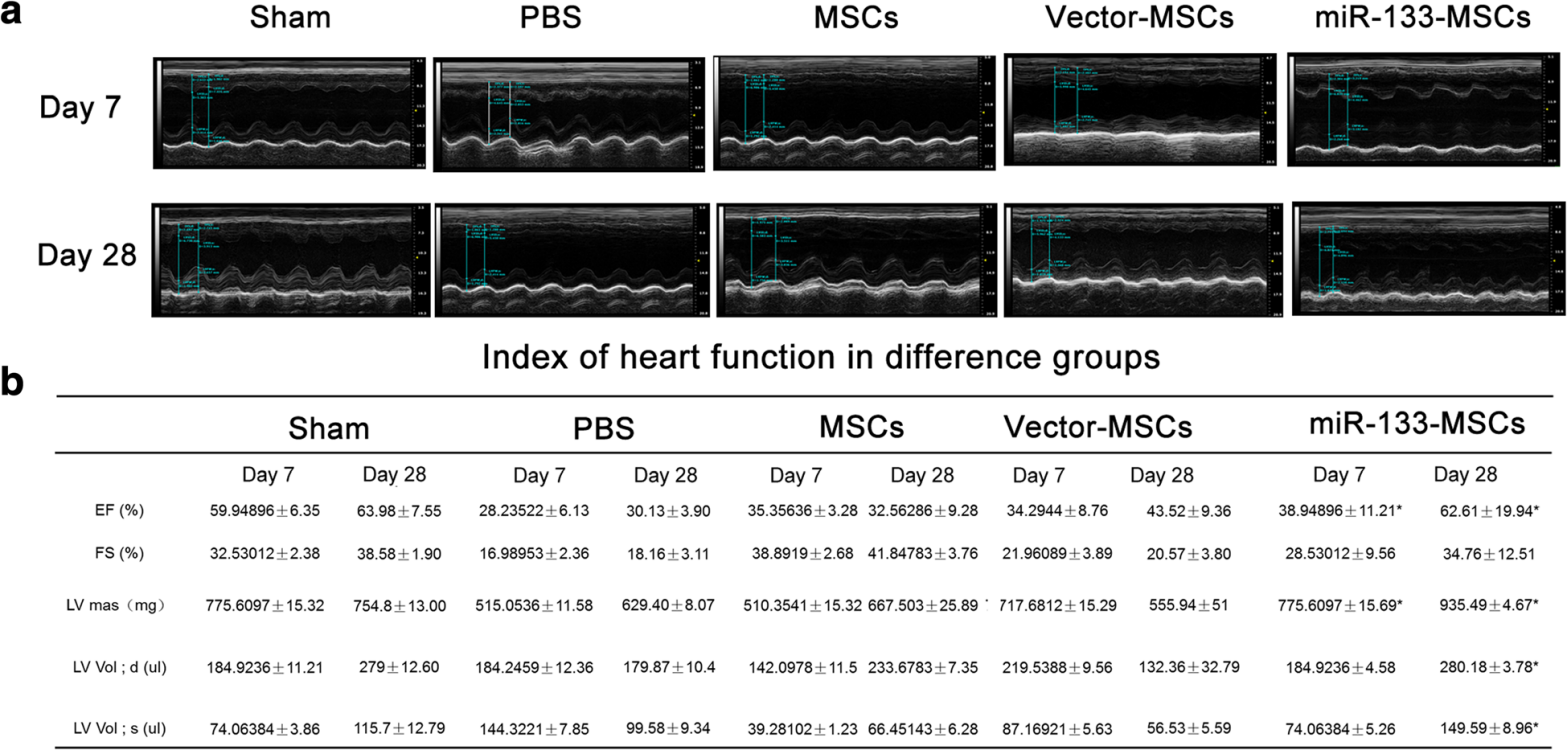
miR-133-MSCs preserve cardiac function. **a** Echocardiogram of rat heart in different groups at 7 and 28 days after MI. **b** Index of heart function in different groups. *p < 0.05 compared with vector-MSC group. EF ejection fraction, FS fractional shortening, LV left ventricular, MSC mesenchymal stem cell, PBS phosphate-buffered saline

We assessed the in-vivo therapeutic effects of miR-133-MSCs on a rat MI model. Echocardiography was performed at baseline and 7 and 28 days after MI. The left ventricular ejection fraction (LVEF) and fractional shortening (FS) were used as the major in-vivo index for heart function. There was no significant difference among the groups before MI (data not shown). At 7 and 28 days post MI, the LVEF and FS of infarcted rat hearts significantly decreased in the PBS group compared with the sham group, while both normal and miR-133-overexpressing MSCs could partially rescue MI-induced decrease of LVEF and FS as shown in Fig. 4. More importantly, the LVEF was significantly increased in the miR-133-MSC group compared with the vector-MSC group (*p* < 0.05). Besides, LV mass and LV volume were also improved by miR-133-MSCs.

### miR-133-MSCs effectively reduce inflammation and fibrosis in the MI model

**Fig. 5:**
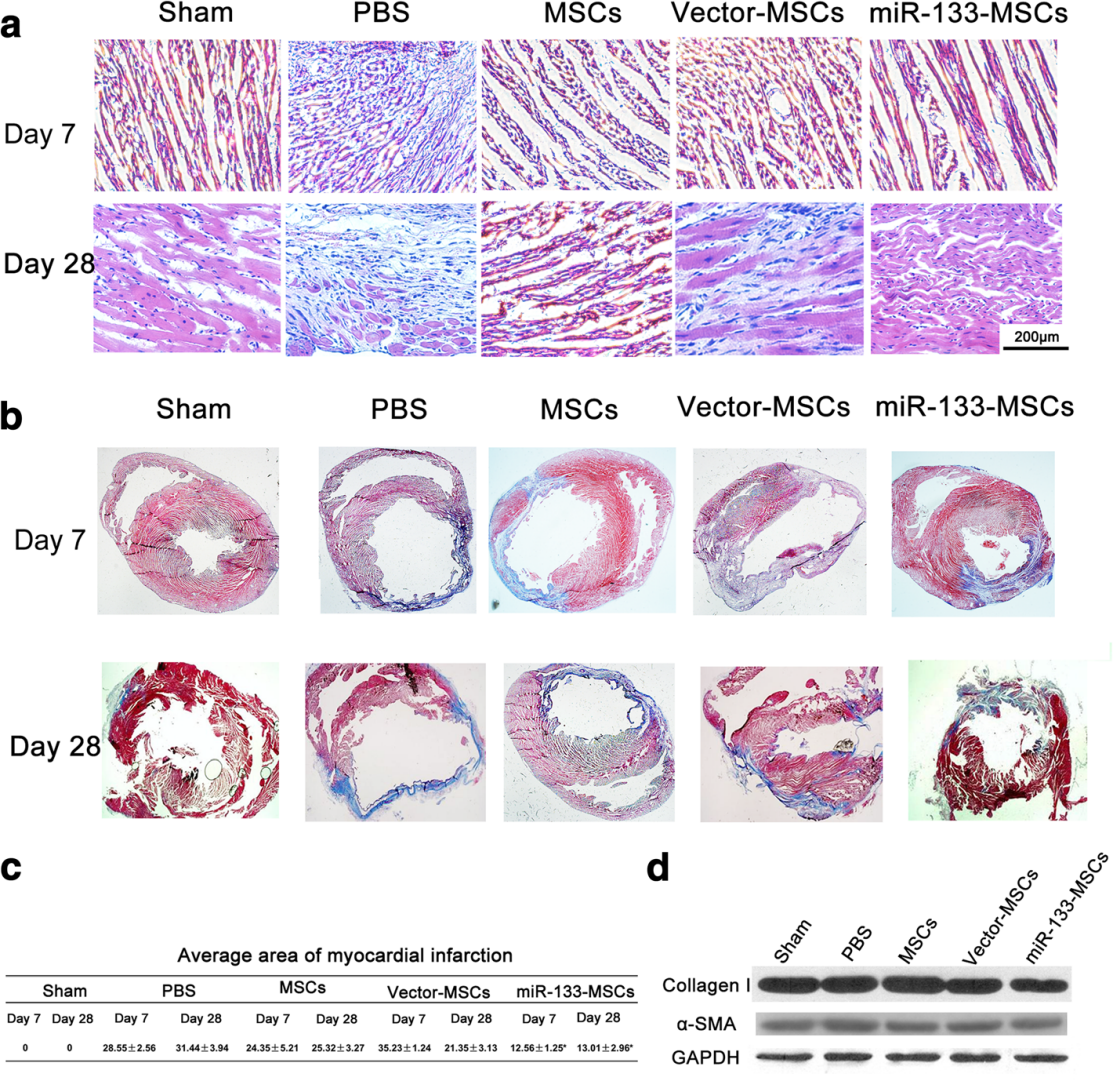
Histological analysis of inflammation and fibrosis in each group. MI was constructed through ligating the left anterior descending artery and then injecting 1 × 10^6^ different MSCs in 20 μl PBS into the peri-infarcted zones. Heart samples harvested 7 or 28 days after cell injection. **a** H&E staining of rat heart sections collected at 7 and 28 days post MI. **b** Masson trichrome staining of heart slides at 7 and 28 days after MI: red, myocardium; blue, scarred fibrosis. **c** Percentage of fibrotic area calculated and averaged by ImageJ software (*n* = 5). **d** Western blot analysis of collagen I and α-SMA protein detected in LV. *p < 0.05 compared with vector-MSC group. MSC mesenchymal stem cell, PBS phosphate-buffered saline

To determine the level of severity, we examined pathology of heart sections 7 and 28 days after MI. Histological assessment of heart slices revealed that cell apoptosis and inflammatory cell infiltration were ameliorated in miR-133-MSC-treated hearts (Fig. 5a).

Following inflammatory cell infiltration, the infarcted heart undergoes fibrosis and remolding to replace necrotic myocardial cells, which leads to further reduction of cardiac function. To evaluate the size of fibrosis, Masson trichrome staining was performed on heart sections 7 and 28 days after MI and MSC intramyocardial injection, respectively. As illustrated in Fig. 5b, normal myocardium was stained red, while the blue color represented fibrosis tissue. Analysis with ImageJ software demonstrated that the fibrosis area in the miR-133-MSC group is significantly reduced compared to both PBS and vector-MSC groups, indicating a better therapeutic effect of miR-133-MSCs on MI (Fig. 5c). Western blot analysis of collagen I and α-SMA further confirmed the fibrosis level in the different groups already described (Fig. 5d).

### miR-133 enhances the survival of MSCs in vivo and reduces the cardiac expression of *Snail 1* in vitro and in vivo

**Fig. 6:**
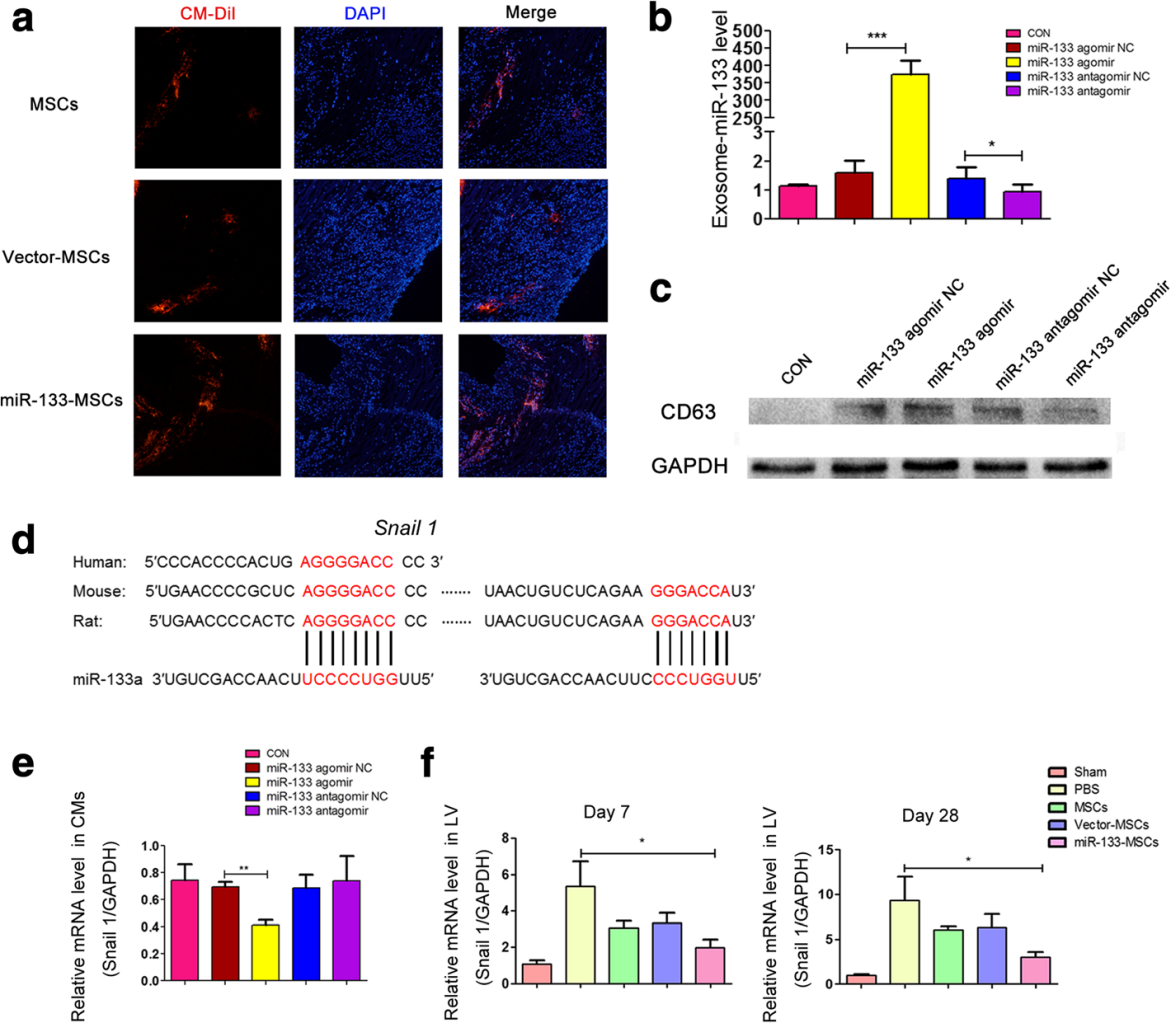
miR-133 enhanced survival of MSCs in vivo and downregulated expression of *snail 1*. Survival of MSCs indicated by CM-Dil (chloromethylbenzamido) and expression level of *snail 1* analyzed by Q-PCR. **a** Fluorescent photographs of surviving MSCs with red fluorescence. **b** miR-133 level in exosome isolated from MSC culture supernatant. **c** Western blot analysis of CD63 in exosome. **d** *Snail 1* 3′UTR contains predicted miR-133 binding sites, which are conserved among species. **e** Relative expression of *snail 1* in cardiomyocytes cocultured with different MSCs. **f** Relative expression of *snail 1* in left ventricular tissue at 7 and 28 days after MI. *p < 0.05, **p < 0.01, ***p < 0.001. CON control, LV left ventricle, MSC mesenchymal stem cell, NC negative control, PBS phosphate-buffered saline

One of the most important barriers to MSC therapy in MI is that many of the MSCs injected into the heart die in the hypoxic environment or are washed away with heart beating. We therefore assessed the survival and retention of MSCs in the heart 3 days after cell injection. While all cells in the LV were stained by DAPI, CM-Dil^+^ cells indicated only the viable exogenous MSCs. The results showed that the miR-133-MSC group possesses more survival MSCs after MI compared with the other two groups (Fig. 6a).

Exosomes derived from MSCs were proved to influence cell apoptosis and angiogenesis potency [28]. To study whether exosomes mediate the roles of miR-133-MSCs in the heart function after MI, the abundance of miR-133 was accessed in exosomes secreted by miR-133-overexpressing or interfering MSCs. Our results demonstrated that the miR-133 level was elevated by 200-fold compared with the negative control (Fig. 6b). Thus, miR-133 secreted from MSCs might influence the biological functions of other cells. Western blot analysis showed that, on the same amount, the exosome isolated from the miR-133 agomir group expressed a high level of CD63, a protein marker in the exosomes (Fig. 6c). We then searched for a potential direct mRNA target of miR-133 by a target prediction program, and identified two conserved miR-133 binding sites in the 3′UTR of the *snail 1* gene (Fig. 6d). *Snail 1* is a master regulator of epithelial-to-mesenchymal transition (EMT), and induces fibrogenesis during developmental and disease processes. By in-vitro coculture assay, we found that miR-133-MSCs significantly repressed the cardiac expression of *snail 1* mRNA (Fig. 6e). Coincidently, while the expression level of *snail 1* was obviously increased in the left ventricle of the infarcted heart at both 7 and 28 days post MI, miR-133-overexpressing MSCs revealed more effective suppression of MI-induced *snail* expression than control-MSC or vector-MSC injection (Fig. 6f).

These results suggested that miR-133 might enhance the survival of MSCs injected into the heart and influence the paracrine function of MSCs including miR-133 and other protein levels, and that miR-133 secreted from MSCs might suppress the expression of *snail 1* in cardiomyocytes in vitro and in vivo and then influence the fibrosis of LV after MI.

## Discussion

Acute myocardial infarction is characterized by ischemic injury and cardiomyocyte cell death, which leads to cardiac dysfunction and eventually causes heart failure. Apoptotic cell death of cardiomyocytes is one of the main types of cardiac death during the process of MI. Although MSC transplantation was considered a potential strategy for MI, most transplanted MSCs undergo cell apoptosis in the ischemic myocardium microenvironment. A large number of studies have revealed important roles of miRNAs in regulating cell proliferation, differentiation, apoptosis, pyroptosis, and inflammatory responses [14, 29–31]. In this study, we revealed a protective function of miR-133 against hypoxia-induced MSC apoptosis, as well as more effectively improved cardiac function in the infarcted heart following transplantation of miR-133-overexpressing MSCs.

Previous studies reported an inconsistent modulatory effect of miR-133 on cell proliferation and differentiation through different signaling pathways [32, 33]. In this study, we found miR-133 had little influence on proliferation of MSCs. We thus explored whether miR-133 could protect MSCs against apoptosis. By the method of hypoxia, a widely used in-vitro model to mimic the ischemic myocardium microenvironment, we first revealed that miR-133 agomir could significantly reduce hypoxia-induced MSC apoptosis at both early (14.89% versus 25.14% in miR-133 agomir NC) and late (2.54% versus 6.15% in miR-133 agomir NC) stages. Consistently, miR-133 antagomir accelerated cell apoptosis of MSCs under hypoxic conditions. Previous data and our data illustrate that miR-133 could be an effective target to promote MSC survival in the ischemic myocardium microenvironment.

Since the survival rate and activity are the two most important determinants of cell function, we assumed that miR-133 overexpression may improve the effect of MSCs on cardiac protection in the injured heart. In this study, we proved that, compared with PBS, MSC, and vector-MSC groups, miR-133-MSCs could ameliorate the symptoms of MI, such as increasing the LVEF and FS, reducing the inflammatory cell infiltration in the heart at 7 and 28 days post MI, and decreasing fibrosis. The improved cardiac function in the miR-133-MSC group was partially caused by increased survival of transplanted MSCs, as indicated by CM-Dil staining.

It is generally believed that the function of MSCs is mediated mostly by paracrine action through soluble factors including miRNAs and proteins [28]. Consistently, our results indicated an increased expression of exosome biomarker CD63 in the miR-133 overexpression group. Compared to the negative control, we further found a significant increase of the miR-133 level in exosomes secreted by miR-133-overexpressing MSCs. These data indicated that, in addition to promoting MSC survival, the improvement of cardiac function in the infarcted heart could be also mediated by miR-133 secreted from miR-133-MSCs. In this regard, miR-133 has been reported to be deregulated in human MI [18] and cardiac hypertrophy, while its overexpression was able to antagonize cardiac apoptosis [34] and protected the heart from ischemic injury in a mouse model of cardiac hypertrophy [23]. Besides, recent studies revealed the ability of miR-133, along with other transcription factors or miRNAs, to induce myocardial transdifferentiation of cardiac fibroblasts through inhibiting the expression of TGF-β signaling or factors which promote fibrosis, such as *snail 1* [35, 36]. Previous studies have proved that miRNAs in MSC-secreted exosome have a therapeutic effect on inflammatory disease, such as sepsis and spinal cord injury [37, 38]. Correspondingly, we demonstrated in this study that miR-133 overexpression ameliorated inflammation and fibrosis in the infarcted heart. In this regard, two fibrosis-related genes *Col1a1* and *snail 1* were predicted targets of miR-133 by TargetScan (http://www.targetscan.org/vert_71/). Our Q-PCR results further verified that overexpression of miR-133 in MSCs significantly repressed cardiac expression of the *snail 1* gene. Thus, miR-133 secreted by miR-133-MSCs may enhance the function of the infarcted heart through reducing inflammation and fibrosis.

## Conclusions

In summary, this study demonstrated that miR-133 enhanced the therapeutic effect of MSCs in an acute MI model, by suppressing the apoptosis of MSCs under hypoxic conditions and mediating their paracrine effects.

## Additional file

Additional file 1: Is Figure S1 showing influence of miR-133 on apoptosis, viability, and proliferation at different times on MSCs. (**A**) Time-dependent change of early and late apoptosis under hypoxic conditions. (**B**) Cell viability of MSCs transfected with miR-133 agomir and antagomir for 6, 12, and 48 h. (**C**) Cell proliferation of MSCs transfected with miR-133 agomir and antagomir for 6, 12, and 48 h (TIF 3763 kb)

## Abbreviations

ALI: Acute lung injury
CFSE: Carboxyfluorescein diacetate succinimide ester
COL1A1: Collagen type I alpha 1 chain
CXCR4: C-X-C motif chemokine receptor 4
FBS: Fetal bovine serum
FS: Fractional shortening
LPS: Lipopolysaccharide
LVEF: Left ventricular ejection fraction
LVESV: Left ventricular end-systolic volume
MI: Myocardial infarction
MSC: Mesenchymal stem cell
PARP: Poly ADP-ribose polymerase
