# Supplementary figures and images for: MicroRNA-133 overexpression promotes the therapeutic efficacy of mesenchymal stem cells on acute myocardial infarction
Source: Stem Cell Res Ther. 2017 Nov 25;8:268. doi: 10.1186/s13287-017-0722-z (PMC5702098; doi:10.1186/s13287-017-0722-z)

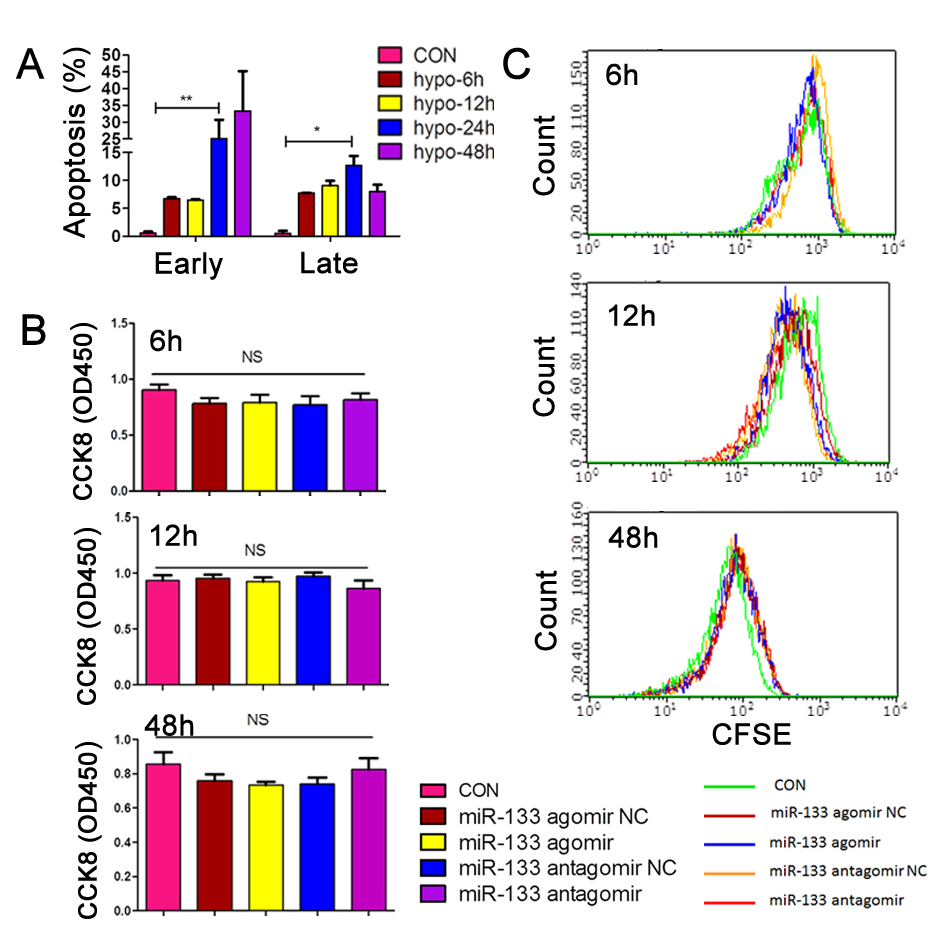

Supplement: Additional file 1: — Is Figure S1 showing influence of miR-133 on apoptosis, viability, and proliferation at different times on MSCs. (A) Time-dependent change of early and late apoptosis under hypoxic conditions. (B) Cell viability of MSCs transfected with miR-133 agomir and antagomir for 6, 12, and 48 h. (C) Cell proliferation of MSCs transfected with miR-133 agomir and antagomir for 6, 12, and 48 h (TIF 3763 kb) [file 13287_2017_722_MOESM1_ESM.tif]
